# Data on the multilocus molecular phylogenies of the Neotropical fish family Prochilodontidae (Teleostei: Characiformes)

**DOI:** 10.1016/j.dib.2016.08.015

**Published:** 2016-08-18

**Authors:** Benjamin W. Frable, Bruno F. Melo, Brian L. Sidlauskas, Kendra Hoekzema, Richard P. Vari, Claudio Oliveira

**Affiliations:** a Department of Fisheries and Wildlife, Oregon State University, Corvallis, OR, USA; b Marine Vertebrate Collection, Scripps Institution of Oceanography, University of California, San Diego, La Jolla, CA, USA; c Departamento de Morfologia, Instituto de Biociências, Universidade Estadual Paulista, Botucatu, São Paulo, Brazil; d Department of Vertebrate Zoology, National Museum of Natural History, Smithsonian Institution, Washington, DC, USA

**Keywords:** Phylogenetics, Prochilodontidae, Characiformes, *BEAST, Shimodaira–Hasegawa test

## Abstract

The data presented herein support the article “Molecular phylogenetics of the Neotropical fish family Prochilodontidae (Teleostei: Characiformes)” (B.F. Melo, B.L. Sidlauskas, B.W. Frable, K. Hoekzema, R.P. Vari, C. Oliveira, 2016) [1], which inferred phylogenetic relationships of the prochilodontids from an alignment of three mitochondrial and three nuclear loci (5279 bp) for all 21 recognized prochilodontid species and 22 related species. Herein, we provide primer sequences, museum voucher information and GenBank accession numbers. Additionally, we more fully describe the maximum-likelihood and Bayesian phylogenetic analyses of the concatenated dataset, detail the Bayesian species tree analysis, and provide the maximum likelihood topologies congruent with prior morphological hypotheses that were compared with the unconstrained tree using Shimodaira–Hasegawa tests.

**Specifications Table**

**Table:** 

Subject area	Biology, Genetics and Genomics
More specific subject area	Phylogenetics and Phylogenomics
Type of data	Tables, figures, primers, sequence alignment, museum voucher information, phylogenetic trees
How data was acquired	DNA extraction from tissue samples, gene amplification, Sanger sequencing
Data format	Raw, filtered, analyzed
Experimental factors	DNA extraction from muscle or fin tissue using Quiagen DNeasy kit or modified NaCl protocol
Experimental features	Sequences concatenated and aligned in Geneious (v.7.1.7), phylogenies generated using unconstrained and constrained maximum-likelihood (RAxML), concatenated Bayesian (MrBayes), and Bayesian species tree (*BEAST) methods.
Data source location	South America
Data accessibility	Data provided with this article and in the GenBank public repository, GenBank: KX086740 through GenBank: KX087100 (see Table 2) and 16S: http://www.ncbi.nlm.nih.gov/popset/1021206184
	COI: http://www.ncbi.nlm.nih.gov/popset/1021205438
	Cytb: http://www.ncbi.nlm.nih.gov/popset/1021205579
	Myh6: http://www.ncbi.nlm.nih.gov/popset/1021205738
	Rag1: http://www.ncbi.nlm.nih.gov/popset/1021205893
	Rag2: http://www.ncbi.nlm.nih.gov/popset/1021206027

**Value of the data**

• New sequence data were used to infer the first complete molecular phylogenetic analysis of family Prochilodontidae.
• Dataset includes DNA sequences for all 21 valid prochilodontid species and 22 related characiform species, many of which are not otherwise represented in Genbank.
• These data facilitate synthesis with previously published sequences and can be reused in other studies because the loci are commonly used in fish phylogenetics.
• Constrained phylogenies permit statistical comparison of new molecular results with prior morphological hypotheses.

## 1 Data

We provide: 1) A table documenting the deposition of museum voucher specimens, 2) aa file containing concatenated alignments for all six loci, 3) a table containing GenBank accession numbers, 4) procedures, parameters and configuration scripts used to estimate phylogenetic relationships, 5) Newick-formatted treefiles inferred with maximum likelihood, concatenated Bayesian, and species tree methods, 6) Newick-formatted treefiles and PDF images of maximum likelihood phylogenies inferred under four topological constraints matching the morphological phylogeny of Castro and Vari [2], and 7) procedures used in Shimodaira–Hasegawa tests of alternative topologies.

## 2 Experimental design, materials and methods

### 2.1 Taxon sampling

This dataset included samples from 77 individuals: 55 individuals representing all 21 species of the three prochilodontid genera, and samples from 22 related taxa from the other three anostomoid families (Anostomidae, Chilodontidae, Curimatidae), three families previously hypothesized to be closely related to Anostomoidea (Hemiodontidae, Parodontidae and Serrasalmidae), and *Brycon pesu* (Bryconidae), as an outgroup. Nine of the samples were derived from previous studies [3], [4], [5], and thus 88% of these data are new to science. We used tissue samples stored in 95% ethanol or a saturated DMSO/NaCl solution, primarily from specimens deposited in museum and university collections (see Table 1 in Melo et al. [1]). We included multiple individuals for each prochilodontid species except *Ichthyoloelephas longirostris*, which is exceedingly rare in tissue collections. The authors BFM, BLS and RPV confirmed the taxonomic identity of most voucher specimens using morphological features.

### 2.2 Molecular dataset

We extracted genomic DNA using DNeasy Tissue kits (Qiagen Inc.) or a modified NaCl protocol from Lopera-Barrero et al. [6]. For this dataset, we amplified partial sequences of the mitochondrial genes *16S rRNA* (16S, 510 bp), *cytochrome oxidase C subunit 1* (COI, 658 bp) and *cytochrome B* (Cytb, 991 bp) using one round of polymerase chain reaction (PCR). Additionally, we acquired sequences of the nuclear *myosin heavy chain 6 gene* (Myh6, 711 bp), *recombination activating gene 1* (Rag1, 1379 bp), and *recombination activating gene 2* (Rag2, 1030 bp) using nested-PCR following Oliveira et al. [3]. Primers for the loci appear in Table 1. We selected these loci as they are commonly used in phylogenetic analyses of Neotropical characiforms [3], [4], [5] and will facilitate subsequent supermatrix analyses and use by other researchers.

Amplification techniques and sequencing reactions are detailed in Melo et al. [1]. We amplified and included all six loci for 42 (of 77) individuals. In the rest of the matrix, we are missing one locus for 22 individuals, two loci for nine individuals, four for one individual and five for three specimens (both specimens of *Ichthyoelephas humeralis* and one of *Prochilodus britskii*; see Table 2). New sequences generated in this analysis were deposited in GenBank with accession numbers KX086740 through KX087100. The precise matches of sequence accession numbers to gene and voucher appear in Table 2.

### 2.3 Alignment, partitioning, and model selection

We aligned and edited sequences using Geneious 7.1.7 ([7]; www.geneious.com). We assigned IUPAC ambiguity codes where we detected uncertainty of nucleotide identity. We performed the alignment of consensus sequences for each gene with the Muscle algorithm [8] implemented in Geneious using default parameters and inspected the sequences visually for obvious misalignments. We estimated the index of substitution saturation (Iss) using Dambe 5.3.38 [9] to evaluate the occurrence of substitution saturation. We found no indication of substitution saturation in transitions or transversions in any topologies. Initial examination of the complete 16S data revealed many uncertain alignments from length polymorphism in loop regions. We excluded these hypervariable regions in a reduced 16S submatrix that was in turn concatenated with the other five genes. The final concatenated dataset for all the sampled taxa is 5279 bp long with 8.9% missing data, 944 (17.9%) identical sites and 1463 of 1970 variable sites being parsimony-informative (matrixfile Prochilodontidae_matrix.nex). Nucleotide frequencies are presented in Table 1.

We used PartitionFinder 1.1.0 [10] to select the partitioning scheme and the model molecular evolution for each partition in the scheme using the Bayesian information criterion (BIC). For this analysis, we assumed 16 possible partitions (Table 3), one for each codon position in the five coding genes (COI, Cytb, Myh6, Rag1 and Rag2), plus the 16S stems. Results identified six partitions with models summarized in Table 3.

### 2.4 Concatenated analyses

We analyzed the partitioned matrix using the Bayesian methods in MrBayes 3.2 [11] with substitution models identified by PartitionFinder (Table 3). We performed two Monte Carlo runs of four independent Markov chains (MCMC) for 20 million generations each, sampling every two thousand replicates. Methods for identifying the maximum-clade credibility (MCC) tree are discussed in Melo et al. [1]. We visualized and edited the final MCC phylogeny with FigTree v1.4.2 (treefile max_cred_tree_newick.nwk).

**Fig. 1:**
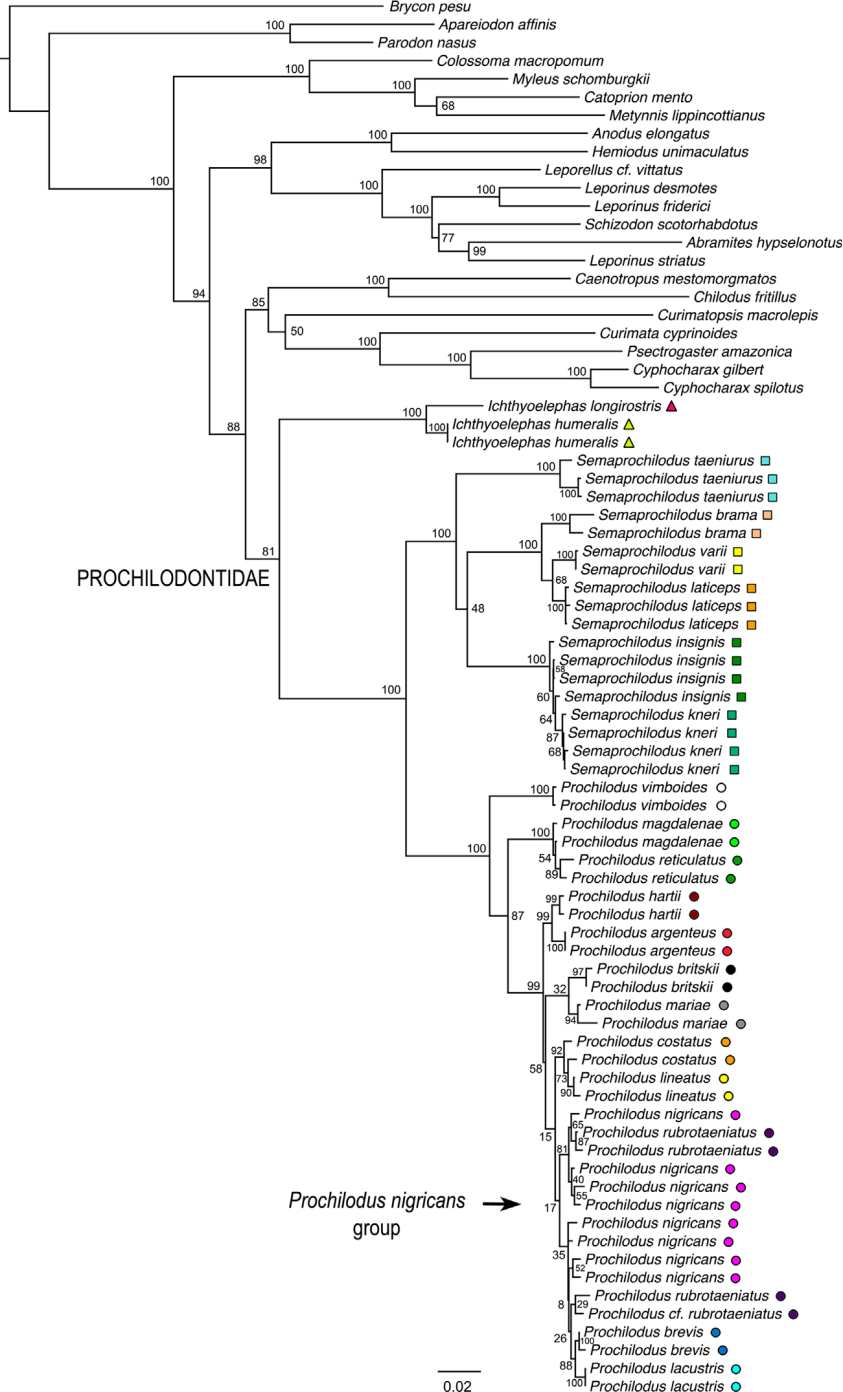
Phylogenetic relationships of Prochilodontidae based on maximum likelihood analysis of the concatenated dataset. Numbers near nodes represent bootstrap support. Colored symbols correspond to those in Fig. 3, Fig. 4 of Melo et al. [1]. (F1_RAxML_bestTree.unconstrained_result.nwk).

We inferred a maximum likelihood (ML) topology using RAxML HPC v.8 on XSEDE [12] on CIPRES Scientific Gateway v.3.3 [13]. Partitioning schemes were identified using PartitionFinder; however, substitution models were restricted to GTR due to the limitations of RAxML. Additional information on the ML analysis is provided in Melo et al. [1]. The final maximum likelihood phylogeny is provided here in treefile RAxML_bipartitions.unconstrained_result (Fig. 1).

### 2.5 Species tree analyses

We implemented the sequence-based species tree ancestral reconstruction method *BEAST [14]. This method estimates the posterior probability of all gene trees and species tree simultaneously from the alignment with informed priors on substitutions and rates of evolution. *BEAST requires *a priori* designation of individuals into species or OTUs (not individual organisms or sequences). Due to the non-monophyletic reconstructions of *Prochilodus nigricans* and *P. rubrotaeniatus* in concatenated analysis (see Melo et al. [1])*,* we assigned those species to two separate species units, denoted by 1 and 2 following the species name (see Fig. 5 in Melo et al. [1]). The final analysis included 77 individuals in 41 nominal species and four taxonomic units. We constrained Prochilodontidae to monophyly based on exceptionally evidence strong from morphology [2], and the concatenated molecular analyses [1]. *Brycon pesu* served as the outgroup.

We hypothesized six possible partitions (one for each gene), and used the BIC in PartitionFinder 1.1.4 [10] to estimate the best partitioning scheme and to select the best-fit model for each gene (Table 4). We implemented the uncorrelated lognormal distribution (UCLN) rate variation model to estimate trees in BEAST v 1.8.3 because previous empirical and simulation studies have demonstrated that the UCLN model is usually the most accurate and robust [15], [16] when local clocks are not expected [17]. A lognormal prior was set on the mean clock rate for each gene (Table 5; BEASTfile StarBeast_Prochilodontidae_250Mgen.xml). A birth-death tree prior was chosen for node time estimation; this models the distribution under a birth-death stochastic branching process model (i.e., speciation and extinction rates can affect a lineage at any time) and is considered the most appropriate when extinction is known or suspected to have occurred in the group [15]. Priors and parameters were set in BEAUti 1.8.3 [18]. We ran four independent MCMC chains for 250 million generations, sampling data every 25,000 generations. The concatenation of the four independent runs attained sufficient coverage after 250 million generations with ESS > 200 for most statistics except for some of the root height priors, which are not as relevant to *BEAST analyses as are divergence time estimates in BEAST. The final maximum clade credibility tree was identified from 32,000 sampled trees with a log clade credibility of −8.56 (Fig. 5 in Melo et al. [1]; treefile StarBeast_MCC_Prochilodontidae_concatenation.nwk).

### 2.6 Shimodaira–Hasegawa tests

**Fig. 2:**
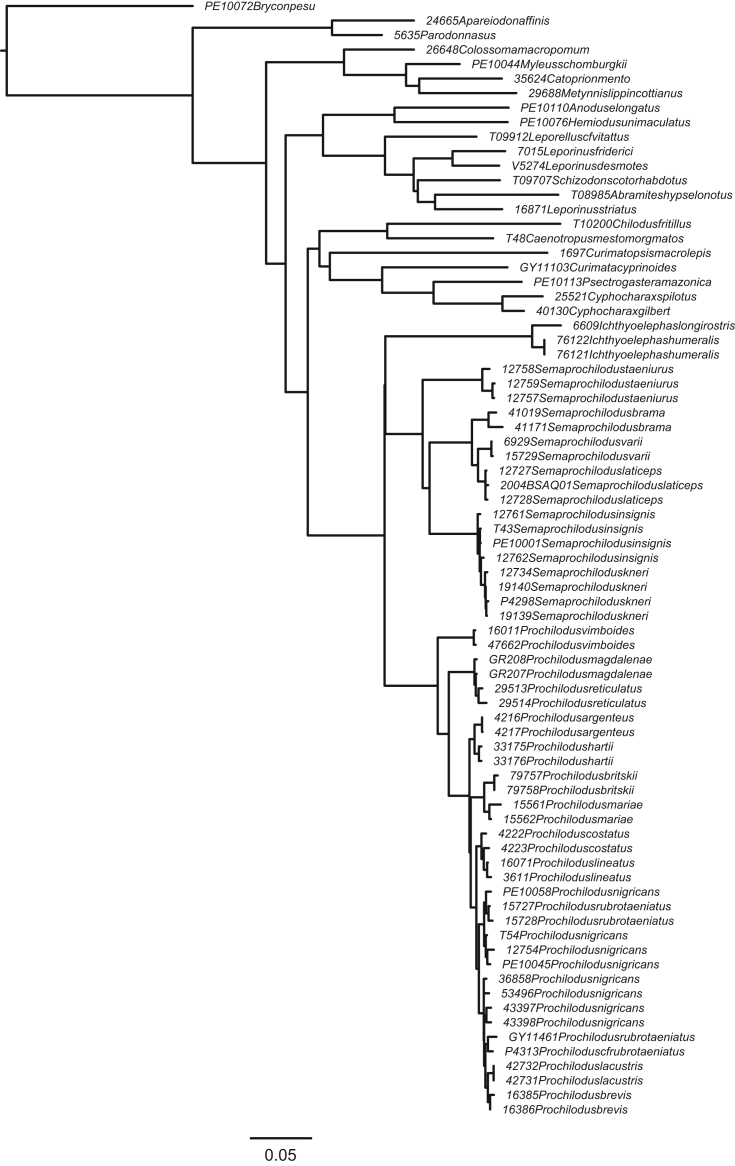
Maximum likelihood topology with *Ichthyoelephas* constrained to be sister to *Semaprochilodus*. (F2_constraint4_Ichthyoelephas_constrained_RAxML_bestTree.result.nwk).

**Fig. 3:**
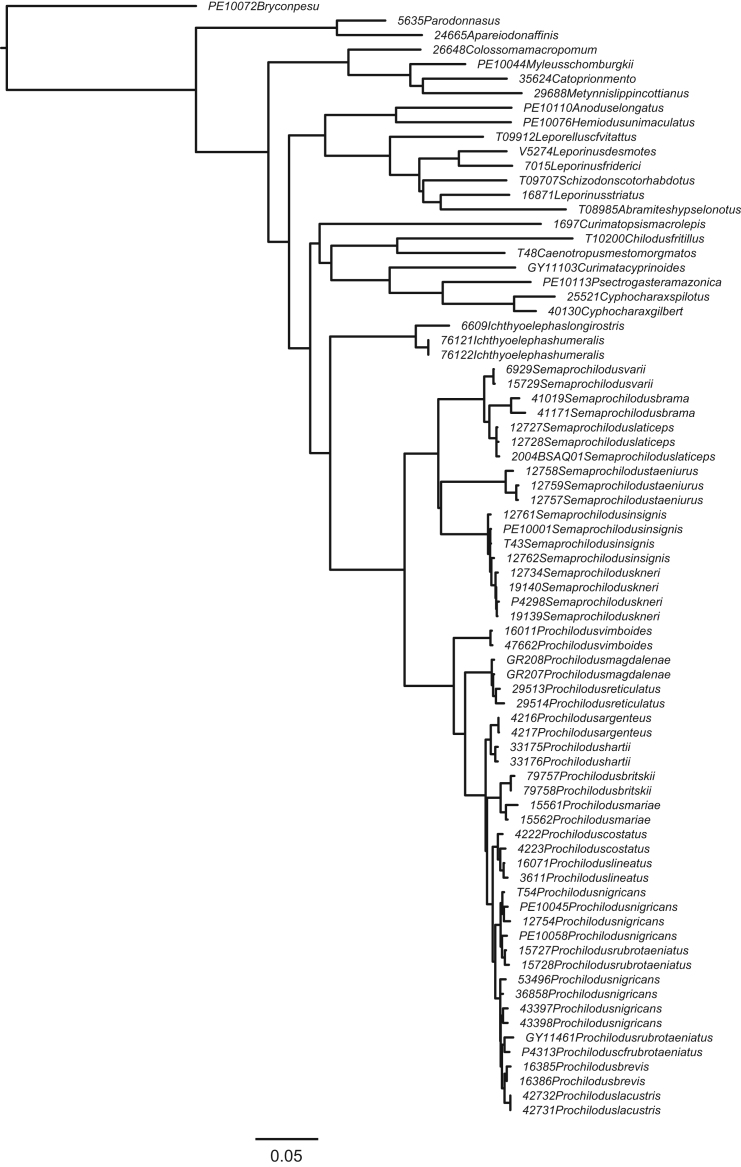
Maximum likelihood topology with *Semaprochilodus taeniurus* constrained to be sister to a clade containing *S. kneri* and *S. insignis*. (F3_constraint1_Semaprochilodus_taeniurus_constrained_RAxML_bestTree.result.nwk).

**Fig. 4:**
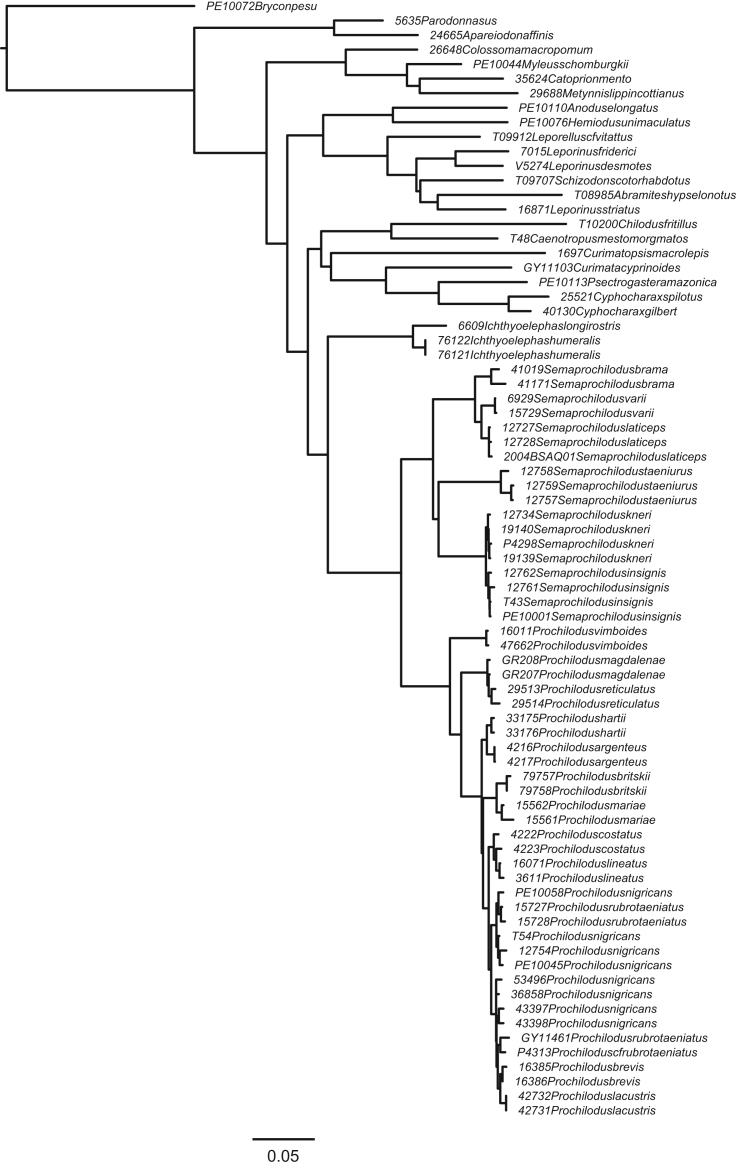
Maximum likelihood topology with *Semaprochilodus taeniurus* constrained to be sister to a clade containing *S. kneri* and *S. insignis*, and *S. insignis* constrained to monophyly. (F4_constraint2_Semaprochilodus_constrained_RAxML_bestTree.result.nwk).

**Fig. 5:**
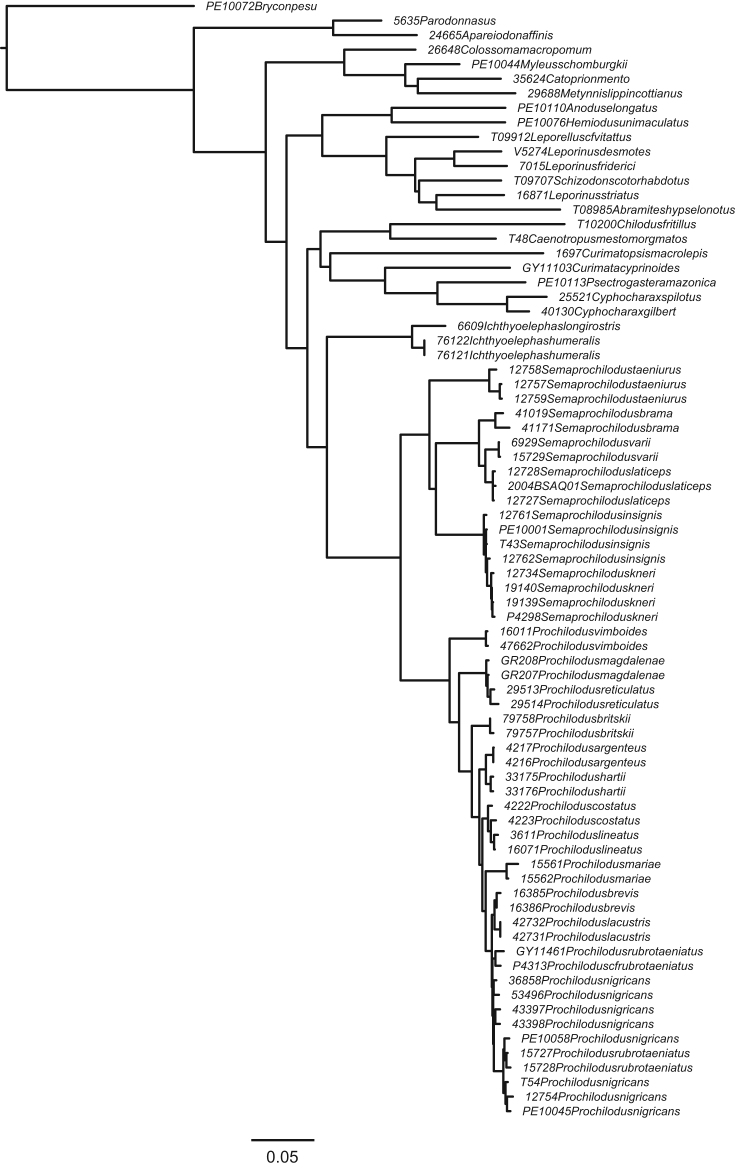
Maximum likelihood topology with intrageneric relationships within *Prochilodus* constrained to those hypothesized by Castro and Vari [2]. (F5_constraint3_Prochilodus_constrained_RAxML_bestTree.result.nwk).

In order to compare support for the most likely molecular topology (Fig. 1; treefile F1_RAxML_bestTree.unconstrained_result.nwk) to support for the morphological hypothesis of Castro and Vari [2], we inferred ML trees in RAxML under four morphology-based constraints discussed in Melo et al. [1]. Constraint trees were created in Mesquite 3.04 [19], and results inferred under those constraints appear in Fig. 2, Fig. 3, Fig. 4, Fig. 5. (treefiles F2_constraint4_Ichthyoelephas_constrained_RAxML_bestTree.result.nwk F3_constraint1_Semaprochilodus_taeniurus_constrained_RAxML_bestTree.result.nwk, F4_constraint2_Semaprochilodus_constrained_RAxML_bestTree.result.nwk, F5_constraint3_Prochilodus_constrained_RAxML_bestTree.result.nwk). The best tree inferred under constraint four (Fig. 2) contains an extremely short branch subtending the *Semaprochilodus* + *Prochilodus* clade, effectively creating a genus-level polytomy. This topology likely results from the much poorer probability of the sequence data given any of the tree models available under constraint four. The maximum likelihood tree under constraint four essentially makes the best of a poor region of parameter space by setting the evolutionary history shared by *Semaprochilodus* and *Ichthyolelephas*, but not *Prochilodus*, to the minimum possible value. Branch length shortening under the other three constraints is substantially more subtle.

We compared the ML unconstrained phylogeny with the four constrained phylogenies using the Shimodaira-Hasegawa (SH) test [20] as implemented in phangorn v2.0.1 [21]. The script for performing these analysis appears here as SHtest.r, and depends upon the FASTA alignment in prochilodontidae.fasta.

## Figures and Tables

**Table 1:** Information content and nucleotide frequencies of each locus.

Locus	Bp after alignment	PCR	Primer sequence (5′–3′)	Π_A_	Π_C_	Π_G_	Π_T_	Reference
16S	510 bp	1 PCR	16Sa-L – ACGCCTGTTTATCAAAAACAT	0.296	0.239	0.236	0.229	[22]
			16Sb-H – CCGGTCTGAACTCAGATCACGT					
								
COI	656 bp	1 PCR	L6252-Asn – AAGCCGGGGAAAGCCCCGGCAG	0.242	0.278	0.187	0.293	[23]
			H7271-COXI – TCCTATGTAGCCGAATGGTTCTTTT					
								
Cytb	990 bp	1 PCR	LNF – GACTTGAAAAACCAYCGTTGT	0.269	0.310	0.146	0.275	[4]
			H08R2–GCTTTGGGAGTTAGDGGTGGGAGTTAGAATC					
								
Myh6	710 bp	1st PCR	F329 – CCGCMTGGATGATCTACACA	0.302	0.229	0.231	0.239	[24]
			3R1 – ATTCTCACCACCATCCAGTTGAA					
		2nd PCR	A3F2 – GGAGAATCARTCKGTGCTCATCA					
			A3R2 – CTCACCACCATCCAGTTGAACAT					
								
Rag1	1378 bp	1st PCR	Rag1CF1 – ACCCTCCGTACTGCTGAGAA	0.250	0.239	0.287	0.224	[4]
			Rag1CR1 – CGTCGGAAGAGCTTGTTGCC					
			Rag1CF2 – TACCGCTGAGAAGGAGCTTC					
		2nd PCR	Rag1CR2 – TGTTGCCAGACTCATTGCCCTC					
								
Rag2	1029 bp	1st PCR	164F – AGCTCAAGCTGCGYGCCAT	0.242	0.259	0.273	0.225	[4], [25]
			Rag2-R6 – TGRTCCARGCAGAAGTACTTG					
			176F – GYGCCATCTCATTCTCCAACA					
		2nd PCR	Rag2Ri - AGAACAAAAGATCATTGCTGGTCGGG					

**Table 2:** Specimens and loci used in Melo et al. [1]. For each individual, its taxonomic designation, collection catalog number of voucher, tissue specimen number, and GenBank accession numbers are given (GenBank:KX086740 through GenBank:KX087100).

Taxon	Voucher	Specimen	16S	Co1	Cytb	Myh6	Rag1	Rag2
*Ichthyoelephas humeralis*	LBP 19,326	76,121	–	–	–	–	–	KX086993
*Ichthyoelephas humeralis*	ANSP 192,865	76,122	–	–	–	–	–	KX086994
*Ichthyoelephas longirostris*	ANSP 192,865	6609	KX087044	–	KX086809	KX086870	KX086956	KX086992
*Prochilodus argenteus*	LBP 251	4216	KX087085	KX086742	KX086841	KX086866	–	–
*Prochilodus argenteus*	LBP 251	4217	KX087086	KX086743	KX086842	KX086867	KX086949	KX087006
*Prochilodus brevis*	LBP 2496	16,385	KX087087	KX086759	KX086829	KX086885	KX086937	KX086995
*Prochilodus brevis*	LBP 2496	16,386	–	KX086760	KX086832	KX086886	KX086938	KX087015
*Prochilodus britskii*	LBP 20,269	79,757	KX087071	–	–	–	–	KX086999
*Prochilodus britskii*	LBP 20,269	79,758	–	–	–	–	–	KX086996
*Prochilodus costatus*	LBP 252	4222	KX087079	KX086744	KX086821	KX086868	KX086950	KX087009
*Prochilodus costatus*	LBP 252	4223	KX087080	KX086745	KX086822	KX086869	–	KX087012
*Prochilodus harttii*	LBP 7211	33,175	KX087098	KX086765	KX086843	–	–	KX087004
*Prochilodus harttii*	LBP 7211	33,176	KX087100	KX086766	KX086844	KX086892	KX086944	KX087005
*Prochilodus lacustris*	LBP 9104	42,731	KX087089	–	KX086830	KX086897	KX086951	KX087017
*Prochilodus lacustris*	LBP 9104	42,732	KX087096	–	KX086831	KX086898	–	KX087018
*Prochilodus lineatus*	LBP 45	3611	KX087081	KX086741	KX086819	KX086865	–	KX087007
*Prochilodus lineatus*	LBP 2348	16,071	KX087082	KX086758	KX086820	KX086884	–	–
*Prochilodus magdalenae*	GR-93-1	GR207	KX087072	KX086779	KX086817	–	KX086959	KX087022
*Prochilodus magdalenae*	GR-93-1	GR208	KX087073	KX086780	KX086818	–	KX086960	KX087023
*Prochilodus mariae*	LBP 2188	15,561	KX087077	KX086755	KX086839	KX086881	KX086931	KX087001
*Prochilodus mariae*	LBP 2188	15,562	KX087078	KX086756	KX086840	KX086882	KX086932	–
*Prochilodus nigricans*	LBP 1690	12,754	–	KX086749	KX086823	KX086875	–	KX087019
*Prochilodus nigricans*	LBP 7841	36,858	KX087088	KX086767	KX086835	KX086893	KX086945	KX087016
*Prochilodus nigricans*	LBP 8589	43,397	KX087084	KX086771	KX086837	KX086899	KX086952	KX087003
*Prochilodus nigricans*	LBP 8589	43,398	KX087076	KX086772	KX086838	KX086900	KX086953	KX087013
*Prochilodus nigricans*	LBP 12,865	53,496	KX087090	KX086774	KX086836	KX086902	KX086955	KX087014
*Prochilodus nigricans*	OS 18,792	PE10045	KX087093	KX086787	KX086827	KX086913	KX086966	KX087000
*Prochilodus nigricans*	OS 18,792	PE10058	KX087094	KX086788	KX086824	KX086914	–	–
*Prochilodus nigricans*	FMNH 113,534	T54	KX087095	KX086797	KX086828	KX086925	KX086974	KX087020
*Prochilodus reticulatus*	LBP 6127	29,513	KX087099	KX086764	KX086816	KX086891	KX086943	KX087021
*Prochilodus reticulatus*	LBP 6127	29,514	HQ171358	KF562435	HQ289647	HQ289067	HQ289260	HQ289453
*Prochilodus* cf. *rubrotaeniatus*	ANSP 40,692	P4313	KX087092	KX086784	KX086834	KX086910	KX086963	KX087002
*Prochilodus rubrotaeniatus*	MHNG 2705.008	SU07108	KX087091	KX086775	KX086825	KX086903	KX086933	KX087010
*Prochilodus rubrotaeniatus*	MHNG 2717.017	SU08776	KX087097	KX086776	KX086826	KX086904	KX086934	KX087011
*Prochilodus rubrotaeniatus*	USNM 403,693	GY11461	KX087083	KX086782	KX086833	KX086908	KX086935	KX087008
*Prochilodus vimboides*	LBP 2349	16,011	KX087075	KX086757	KX086814	KX086883	KX086936	KX086997
*Prochilodus vimboides*	LBP 10,180	47,662	KX087074	KX086773	KX086815	KX086901	KX086954	KX086998
*Semaprochilodus brama*	LBP 12,776	41,019	KX087069	KX086769	KX086856	KX086895	KX086947	KX087029
*Semaprochilodus brama*	LBP 12,807	41,171	KX087070	KX086770	KX086857	KX086896	KX086948	KX087031
*Semaprochilodus insignis*	LBP 1692	12,761	KX087063	KX086753	KX086850	KX086879	–	KX087032
*Semaprochilodus insignis*	LBP 1692	12,762	KX087064	KX086754	KX086849	KX086880	KX086929	–
*Semaprochilodus insignis*	OS 18,380	PE10001	KX087067	KX086785	KX086851	KX086911	KX086964	KX087033
*Semaprochilodus insignis*	ANSP 180,205	T43	KX087061	KX086796	KX086852	KX086923	KX086973	KX087034
*Semaprochilodus kneri*	LBP 1384	12,734	KX087062	–	KX086845	KX086874	KX086928	KX087035
*Semaprochilodus kneri*	LBP 3041	19,139	KX087065	–	KX086846	KX086888	KX086941	KX087036
*Semaprochilodus kneri*	LBP 3041	19,140	KX087066	KX086762	KX086848	KX086889	–	–
*Semaprochilodus kneri*	ANSP 187,277	P4298	KX087060	KX086783	KX086847	KX086909	KX086962	KX087037
*Semaprochilodus laticeps*	LBP 1383	12,727	KX087059	KX086748	KX086861	KX086873	KX086927	–
*Semaprochilodus laticeps*	LBP 1383	12,728	HQ171245	KF562436	HQ289536	HQ288955	HQ289152	HQ289343
*Semaprochilodus laticeps*	FMNH 113,712	2004BSAQ01	KX087068	KX086778	KX086860	KX086906	KX086942	KX087030
*Semaprochilodus taeniurus*	LBP 1691	12,757	KX087051	KX086750	KX086854	KX086876	–	KX087025
*Semaprochilodus taeniurus*	LBP 1691	12,758	KX087050	KX086751	KX086853	KX086877	–	KX087024
*Semaprochilodus taeniurus*	LBP 1691	12,759	KX087052	KX086752	KX086855	KX086878	–	KX087026
*Semaprochilodus varii*	MHNG uncatalogued	15,729	KX087058	KX086777	KX086859	KX086905	KX086930	KX087027
*Semaprochilodus varii*	ANSP 187,435	6929	KX087057	KX086746	KX086858	KX086871	KX086957	KX087028
*Leporellus* cf. *vittatus*	AUM 54,212	T09912	–	KX086795	KX086801	KX086921	KX086972	KX086987
*Leporinus desmotes*	AUM 43,700	V5274	KX087040	KX086798	KX086813	KX086926	KX086975	KX086986
*Leporinus friderici*	ANSP 189,264	7015	KX087039	KX086747	KX086812	KX086872	KX086958	KX086985
*Leporinus striatus*	LBP 3180	16,871	KX087048	KX086761	KX086811	KX086887	KX086939	KX086982
*Abramites hypselonotus*	AUM 53,775	T08985	KX087045	KX086793	KX086808	KX086919	KX086970	KX086981
*Schizodon scotorhabdotus*	AUM 53,654	T09707	KX087047	KX086794	KX086810	KX086920	KX086971	KX086984
*Chilodus fritillus*	AUM 51,355	T10201	KF562391	KF562418	KX086863	KX086922	KF562495	KX086988
*Caenotropus mestomorgmatos*	ANSP 180,516	T48	KF562384	KF562412	KF562442	KX086924	KF562490	KX086991
*Curimatopsis macrolepis*	ANSP 178,188	1697	KX087053	KX086740	KX086800	KX086864	KX086940	KX086977
*Curimata cyprinoides*	USNM 402,471	GY11-1-03	KX087054	KX086781	KX086803	KX086907	KX086961	KX086978
*Psectrogaster amazonica*	OS 18,313	PE10113	KX087049	KX086792	KX086802	KX086918	KX086969	KX086990
*Cyphocharax gilbert*	LBP 8343	40,130	KX087056	KX086768	KX086805	KX086894	KX086946	KX086989
*Cyphocharax spilotus*	LBP 4747	25,521	KX087055	KX086763	KX086804	KX086890	–	–
*Anodus elongatus*	OS 18,724	PE10110	KX087043	KX086791	KX086806	KX086917	–	KX086983
*Hemiodus unimaculatus*	OS18345	PE10076	KX087042	KX086790	KX086807	KX086916	KX086968	KX086980
*Apareiodon affinis*	LBP 4591	24,665	HQ171328	–	HQ289617	HQ289037	HQ289230	HQ289424
*Parodon nasus*	LBP 1135	5635	HQ171429	–	HQ289714	HQ289137	HQ289328	HQ289521
*Colossoma macropomum*	LBP 5173	26,648	HQ171343	–	HQ289632	HQ289052	HQ289245	HQ289438
*Catoprion mento*	LBP 7556	35,624	HQ171392	–	HQ289679	HQ289100	HQ289293	–
*Metynnis lippincottianus*	LBP 6282	29,688	KX087041	–	HQ289651	HQ289072	HQ289265	HQ289458
*Myleus schomburgkii*	OS 18,990	PE10044	KX087046	KX086786	KX086862	KX086912	KX086965	KX086979
*Brycon pesu*	OS 18,361	PE10072	KX087038	KX086789	KX086799	KX086915	KX086967	KX086976

**Table 3:** Position of each gene and codon within the alignment, with their partitions and best models of nucleotide evolution as determined by PartitionFinder.

Gene	Position	Partition	Best BIC model for MrBayes
16S	1–510	1	SYM+I+G
COI 1st position	511–1167/3	2	GTR+G
COI 2nd position	512–1167/3	1	SYM+I+G
COI 3rd position	513–1167/3	3	HKY+I+G
Cytb 1st position	1169–2158/3	4	GTR+G
Cytb 2nd position	1170–2158/3	1	SYM+I+G
Cytb 3rd position	1168–2158/3	3	HKY+I+G
Myh6 1st position	2160–2869/3	6	HKY+I+G
Myh6 2nd position	2161–2869/3	6	HKY+I+G
Myh6 3rd position	2159–2869/3	5	SYM+G
Rag1 1st position	2871–4248/3	6	HKY+I+G
Rag1 2nd position	2872–4248/3	6	HKY+I+G
Rag1 3rd position	2870–4248/3	5	SYM+G
Rag2 1st position	4249–5278/3	6	HKY+I+G
Rag2 2nd position	4250–5278/3	6	HKY+I+G
Rag2 3rd position	4251–5278/3	5	SYM+G

**Table 4:** Partitioning schemes and substitution models for *BEAST identified using the Bayesian Information Criterion in PartitionFinder.

Gene	Position	Partition	Best BIC model for *BEAST
16S	1–510	1	SYM+I+G
COI	511–1167	2	GTR+I+G
Cytb	1169–2158	2	GTR+I+G
Myh6	2159–2869	3	TrNef+I+G
Rag1	2870–4248	3	TrNef+I+G
Rag2	4249–5278	3	TrNef+I+G

**Table 5:** Prior parameter settings for major priors applied in *BEAST. Prior names as in *BEAST/Beauti and are described in BEAST documentation [18].

Prior	Distribution	Initial	Mean/Shape	Scale	Standard deviation	Offset	Upper	Lower
Species.popMean	Gamma	1	1.6	0.5	–	0	–	–
BirthDeath.meanGrowthRate	Uniform	0.8	–	–	–	–	10,000	0
BirthDeath.relativeDeathRate	Uniform	0.5	–	–	–	–	1	0
16S.ucld.mean	–	–	–	–	–	–	–	–
16S.ucld.stdev	Lognormal	0.333	0.5	–	–	0	–	–
COXI.ucld.mean	Lognormal	0.003	0.003	–	1	0	–	–
COXI.ucld.stdev	Lognormal	0.333	0.5	–	–	0	–	–
CYTB.ucld.mean	Lognormal	0.003	0.003	–	1	0	–	–
CYTB.ucld.stdev	Lognormal	0.333	0.5	–	–	0	–	–
MYH6.ucld.mean	Lognormal	0.0005	0.0005	–	1	0	–	–
MYH6.ucld.stdev	Lognormal	0.333	0.5	–	–	0	–	–
RAG1.ucld.mean	Lognormal	0.0005	0.0005	–	1	0	–	–
RAG1.ucld.stdev	Lognormal	0.333	0.5	–	–	0	–	–
RAG2.ucld.mean	Lognormal	0.0005	0.0005	–	1	0	–	–
RAG2.ucld.stdev	Lognormal	0.333	0.5	–	–	0	–	–
